# Evaluation of CNTs and SiC Whiskers Effect on the Rheology and Mechanical Performance of Metakaolin-Based Geopolymers

**DOI:** 10.3390/ma15176099

**Published:** 2022-09-02

**Authors:** Madeleing Taborda-Barraza, Francine Padilha, Laura Silvestro, Afonso Rangel Garcez de Azevedo, Philippe Jean Paul Gleize

**Affiliations:** 1 Laboratory of Application of Nanotechnology in Civil Construction (LabNANOTEC), Department of Civil Engineering, Federal University of Santa Catarina (UFSC), Florianópolis 88040-900, Brazil; 2 Civil Engineering Coordination, Federal University of Technology-Paraná (UTFPR), Guarapuava 85053-525, Brazil; 3 LECIV—Civil Engineering Laboratory, UENF—State University of the Northern Rio de Janeiro, Campos dos Goytacazes 28013-602, Brazil

**Keywords:** geopolymer, metakaolin, nanomaterial, whiskers, rotational rheometry, compressive strength, kinetics

## Abstract

Despite geopolymers having emerged as a more sustainable alternative to Portland cement, their rheological properties still need to be thoroughly investigated, aiming at the material’s applicability. Additionally, studies that evaluated the fresh state of geopolymer composites with nanomaterials are scarce. Thus, two metakaolin-based geopolymer systems were reinforced with nanomaterials with a similar geometry: carbon nanotubes (CNT) and silicon carbide whiskers (SCW). The nanomaterials incorporation was assessed by rotational rheometry (conducted up to 110 min), isothermal calorimetry, compressive strength after 7 and 28 days, and the microstructure was investigated using X-ray diffraction (XRD) and Fourier-transform infrared spectroscopy (FTIR). CNT and SCW incorporation (0.20 wt.%) did not significantly affect the yield stress and viscosity of the R2-group (based on metakaolin type 2), while increasing the rheological parameters up to 56.0% for the R1-group (based on metakaolin type 1). Both additions modified the reaction kinetics. Increments of up to 40.7% were observed in the compressive strength of geopolymer pastes with the incorporation of a SCW content of 0.2 wt.%. XRD and FTIR results suggest similar structural modifications between precursors. Nevertheless, R2 showed substantial transformations while the R1 group exhibited anhydrous material that can react over time. Overall, incorporating CNT and SCW contributed to higher mechanical increments on systems with average mechanical strength (R1) compared to systems with higher potential mechanical performance (R2).

## 1. Introduction

Incorporating nanomaterials in cementitious and geopolymer matrices is a constant practice that mainly intends to enhance mechanical performance [1,2,3,4,5,6,7] or even confer new thermal, optical, and electrical properties [8,9,10,11]. The fact is that the effects of nanomaterials addition are also conditioned by their good dispersion [11,12]. Once this is achieved, the high cost of these materials is superimposed by the expected technical performance.

Overall, carbon nanotubes (CNT) are hydrophobic [13]. However, covalent functionalization for the insertion of carboxyl groups on their surface increases the hydrophilicity of CNT, improving their dispersion in water [14]. Silicon carbide whiskers (SCW) are not hydrophobic. Still, part of their surface ends up in carbon atoms making it hydrophobic, while the silicon part can couple to water molecules and dissociate them. So, depending on its surface, it can be temporarily dispersed in water [15,16].

Often nanomaterials are added in cementitious and polymeric matrices, and their effects are evaluated based on applications or preliminary studies. Nevertheless, little attention is paid to the characteristics of the matrix in which they will be placed. General nucleation or filler effects are attributed to the nanomaterials without stipulating statements about the nature and characteristics of the matrices, thus not elucidating whether the effect may be more noticeable in one type or another of matrix and the causes associated with it [17,18]. Although nanomaterials are expensive, the quantity incorporated into systems usually did not exceed 5%. In this case, the nanoparticles can act as complementary material on cementitious materials and be applied in situations that require high mechanical strength.

Composites are commonly developed by combining two or more constituents to form a new material with new or different unitary properties. Thus, various combinations can be made: metallic, polymeric, or ceramic matrices with additions of different natures. Composites’ importance is based on their intended applications, as they are technologically strategic materials [19,20,21]. When the added material has a nanometric size and can produce properties different from its macro size [17] the composite is called a nanocomposite. We can find polymer and ceramic matrix components reinforced with particles or fibers in the construction industry. However, polymeric nanocomposites are more scarce than ceramic ones.

To understand the physics and chemistry of interaction between the components, allowances were necessary to consider that geopolymers act like ceramic/cementitious materials. Thus, many examples are references to nanocomposite ceramics.

Geopolymeric matrices have been standing out as a possible alternative to Portland cement (PC) for the reduction in carbon dioxide (CO_2_) emissions, currently responsible for approximately 9.0% of the world’s total emissions [22]. In addition to the high percentage of CO_2_ emissions attributed to PC production, there is a worldwide pressure to achieve carbon neutrality (i.e., zero carbon emissions) [23]. A solid literature review indicated that the equivalent CO_2_ emissions of geopolymer matrices usually are lower than for PC, considering a comparable compressive strength [24]. In this context, the mechanical properties of geopolymer matrices have already been extensively studied [25,26,27,28,29].

Specifically, regarding geopolymeric systems, the fresh-state evaluation and rheological characterization are still limited [30]. Additionally, there is a knowledge gap concerning the time-resolved rheological properties of geopolymers with carbon nanotubes incorporation [31], like other types of nanomaterials (e.g., nano-ZnO, SiC nanowires, graphene). Thus, this study aimed to evaluate the CNT and SCW addition on metakaolin-based geopolymers’ rheological and mechanical properties. Two precursors were evaluated (MK1 and MK2) to understand nanomaterials’ reinforcement capacity as a function of the characteristics of the geopolymer matrix.

## 2. Materials and Methods

### 2.1. Precursors

The metakaolin MK1 was obtained calcinating kaolin at 800 °C for 1 h (KK260, Linn Elektro Therm GmbH, Bad Frankenhausen, Germany) in a static muffle. This calcination temperature was defined based on previous studies using the same kaolin [32,33]. The metakaolin MK2, a commercial product, was provided by a local industrial company. The chemical composition of MK1 and MK2 are reported in Table 1, both obtained by the energy dispersive method (EDX-7000, Shimadzu, Tokyo, Japan). The specific surface area (SSA) was determined using Autosorb-1 (Quantachrome Instruments, Boynton Beach, FL, USA), with N_2_ sorption and the Brunauer–Emmett–Teller (BET) method.

**Figure 1:**
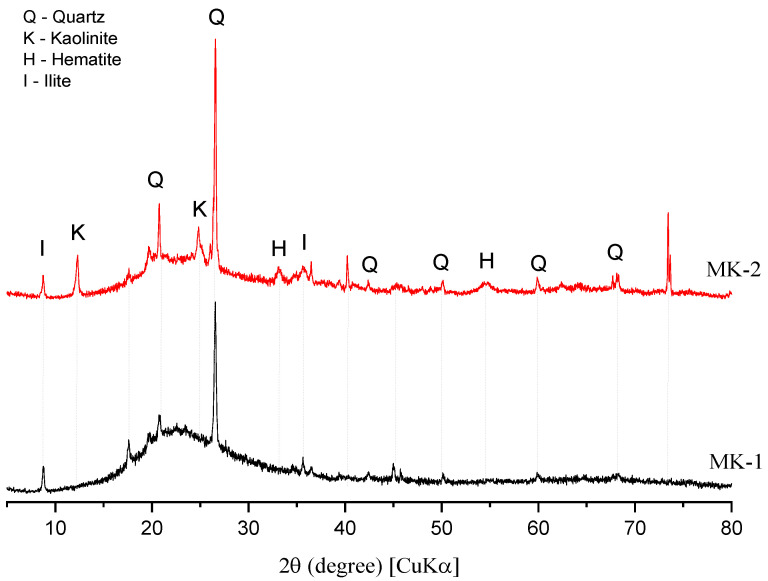
XRD spectra of MK1 and MK2.

The X-ray spectra of both precursors (MK1 and MK2) are reported in Figure 1. The mineralogical analysis indicated kaolinite, hematite, and quartz. Moreover, MK2 is also composed by small amounts of illite. MK1 also showed a higher amorphous halo compared to MK2, which suggests a possible higher reactivity, as further discussed.

**Figure 2:**
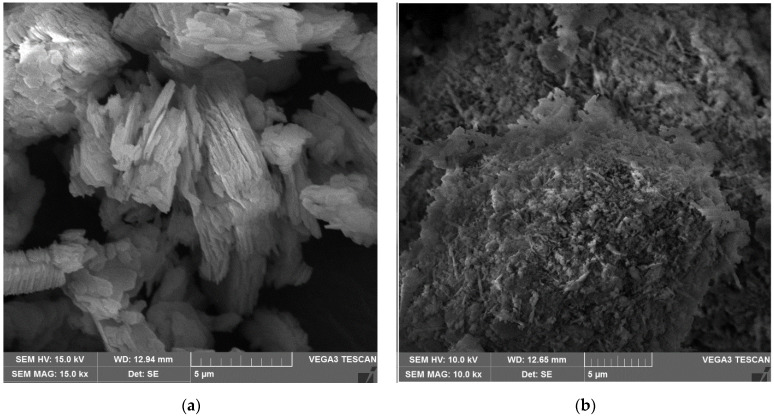
SEM image on: (**a**) MK1; (**b**) MK2 [×15,000].

The precursors MK1 and MK2 were also characterized by scanning electron microscopy (SEM). The samples were stored in a vacuum desiccator, and the analysis was performed using VEGA3 microscopy (TESCAN, Brno, Czech Republic). The geometry of precursors was identified through SEM image, as shown in Figure 2. MK1 looks like stacked units of hexagonal shape, typical of metakaolin, as some authors previously reported [34,35]. MK2 exhibited a rounded shape with an irregular surface, which could get with flash calcination [36].

**Figure 3:**
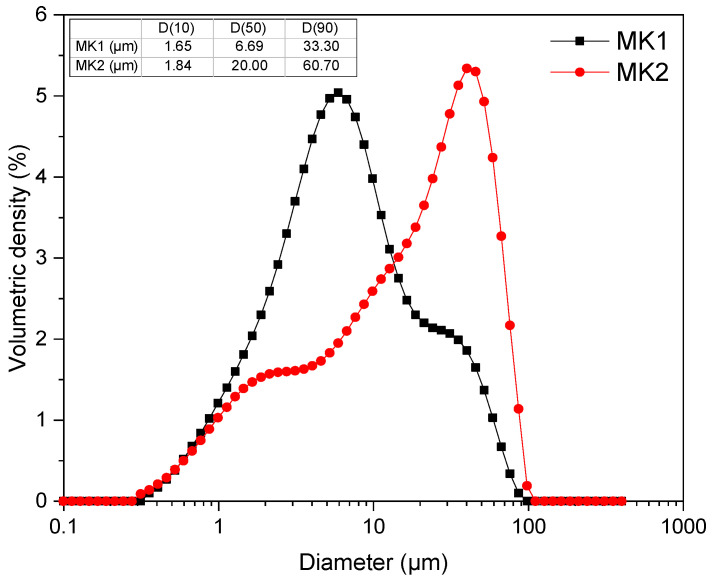
Particle size distribution (PSD) of MK1 and MK2.

Particle size distribution was assessed using the 3500 Particle Size Analyzer (Microtrac, York, PA, USA), with a detection range of 0.1–3500 µm. The particle size distribution (PSD) of both materials is represented in Figure 3, and the specifications of D(10), D(50), and D(90) are indicated in the inserted table. Overall, MK2 has larger particles than MK1, with an average diameter of 20 µm.

**Figure 4:**
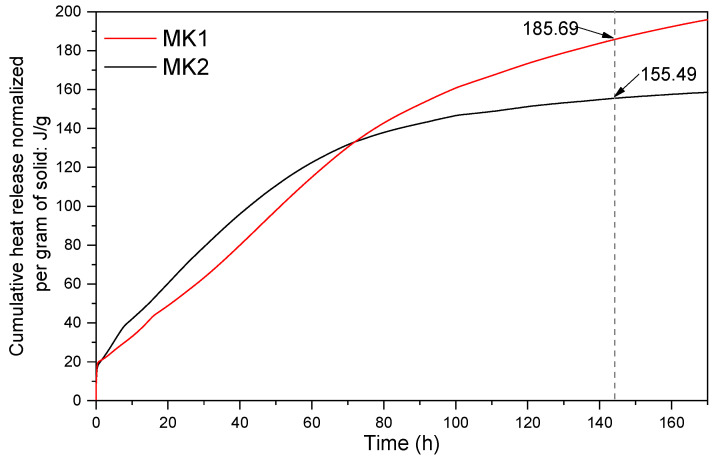
R^3^ test of MK1 and MK2 to assess the reactivity of precursors.

An approach to assess the reactivity of calcined clays is the method proposed by Avet et al. [37], designated as R^3^ test (rapid, relevant, and reliable test). This technique evaluates the reactivity of calcined clay as a function of the heat released after 144 h. Thus, the higher the heat released, the higher the reactivity. This method was adopted to indicate the reactivity of the two precursors evaluated in this study. Initially, the precursor was added to the standard solution, simulating the cement reactions environment. This solution is composed of potassium sulfate (K_2_SO_4_), calcium hydroxide [Ca(OH)_2_], potassium hydroxide (KOH), and water. After adding the precursor to the standard solution, the sample was put into an isothermal TAM Air calorimeter (TA Instruments, New Castle, DE, USA). The cumulative heat release was measured. Considering that the medium to be evaluated is the same for both systems, this technique becomes a comparative test. The results obtained in R^3^ test for MK1 and MK2 are registered in Figure 4 After 144 h, MK1 and MK2 showed a heat release of 185.69 J/g and 155.49 J/g, respectively. These results suggest a higher reactivity of MK1 compared to MK2. Furthermore, it was noted that during the initial period (0–60 h), MK2 was slightly more reactive than MK1, but it immediately got stability in its cumulative heat. In contrast, the heat released by MK1 continues to increase slowly and does not get stability until 170 h.

The reactivity of the precursor is conditionate to calcined temperature and particle size [38]. Moreover, previously mentioned parameters can affect the specific surface area and, consequently, the pozzolanic activity of the SCM [34]. Thus, the SSA of MK1 is approximately 235% higher than MK2 (see Table 1), which is consistent with the higher reactivity of MK1 observed through the R^3^ test.

### 2.2. Alkaline Solution (A.S)

The alkaline activator was prepared with sodium silicate solution (Na_2_SiO_3_–H_2_O), with SiO_2_/Na_2_O = 3.37, and sodium hydroxide—NaOH (micro pellets) (≥98% purity).

### 2.3. Additions

Silicon Carbide Whiskers (SCW), and Carboxyl-Functionalized Multi-Walled Carbon Nanotube (COOH-MWCNT), designated only as CNT, were purchased from Nanostructured & Amorphous Materials Inc. (Katy, TX, USA). Their principal characteristics are shown in Table 2 and Table 3, respectively. The manufacturer provided the dimensions, but the SSA was assessed with the same equipment and conditions described in Section 2.1.

**Figure 5:**
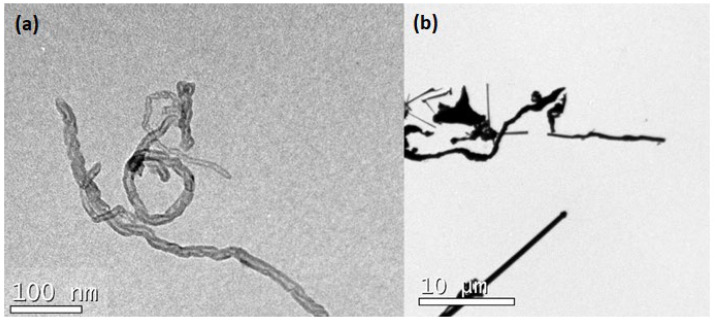
TEM image of: (**a**) CNT; and (**b**) SCW.

The geometry of SCW and CNTs were identified by transmission electron microscopy (TEM, JEM-1011, Joel, Akishima, Japan). The samples were dispersed in water, deposited under a carbon grid, and air-dried until the analysis. Figure 5 shows the irregular shape of nanomaterials. Moreover, note the order of magnitude of the image scales; while CNT has a nanometric scale, SCW has a micrometric scale.

The zeta potential of CNT and SCW aqueous dispersions (concentration of 0.01 wt.%) was measured at a temperature of 25 °C with a pH range of 6 to 12 in a Zetasizer Nano (Malvern, UK), with a measuring range of 3.8 nm to 100 µm. The dispersions were prepared by sonication (see conditions in item 2.4). The pH was adjusted with hydrochloric acid and sodium hydroxide solutions.

### 2.4. CNT and SCW Dispersion

For CNT and SCW dispersion, an ultrasonic energy was applied using a tip sonicator (model VCX 750 W, Sonics & Materials Inc., Newtown, CT, USA). This equipment keeps a frequency of 20 kHz. The dispersion procedure was configurated with an amplitude of 80% (as intensity), duration of 6 min, and cycles of 20s active and 20s resting (to prevent overheating of the suspension) [39]. A cold bath was used to refresh the aqueous dispersions and avoid overheating. These parameters were defined by a group of researchers who identified that a homogeneous dispersion is guaranteed [33].

### 2.5. Samples Preparation

**Figure 6:**
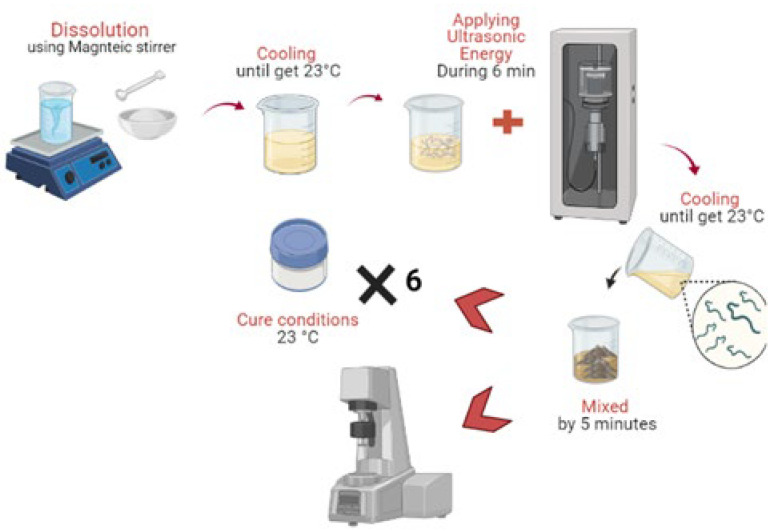
Scheme of experimental procedure for mix preparation.

The A.S. (120.0 g SS and 8.9 g HS), containing (or not) nanomaterials, was added gradually to MK (100.0 g) and mixed for 5 min in a higher-shear mixer (10,000 rpm). The research group defined this proportion for activated metakaolin [33]. Subsequently, all samples were sealed with plastic films and cured at 23 ± 2 °C. The samples were kept at ambient temperature until the day of the tests. Figure 6 shows a scheme of proceeding to the mixing of the materials. Briefly, the NaOH micro pellets were dissolved in a sodium silicate solution using a magnetic stirrer. After the A.S. reached an ambient temperature, the additions (CNT or SCW) were added and dispersed in a probe sonicator for 6 min. Subsequently, the precursors were added to A.S. and mixed for 5 min to prepare the geopolymer pastes. In the fresh state the geopolymers were assessed in a rotational rheometer and added in vials for isothermal conduction calorimetry. For the compressive strength, cylinder specimens were molded (see item 2.6). Table 4 indicates the mix proportions evaluated. A CNT and SCW content of 0.2% related to precursors’ weight was assessed. This content was defined based on research on CNT and SCW incorporation in geopolymer-based matrices [33,40].

### 2.6. Test Methods

The geopolymer pastes’ shear stress and apparent viscosity were measured using a Haake Mars III Rheometer (Thermo Fisher Scientific, Waltham, MA, USA) and a vane rotor FL 22 into a container cylinder. The analysis was conducted with a gap of 11 mm and a standard temperature of 23 °C. The measurement conditions were as follows: pre-shear at 100 s^−1^ for 30 s, after increasing the shear rate from 0.10 s^−1^ to 100 s^−1^ in 200 s, followed by a decrease from 90 to 0.10 s^−1^ in 180 s. This cycle was repeated 5 times, with 20 min of interval, i.e., the shear stress and apparent viscosity were registered at 10, 30, 50, 70, 90, and 110 min. The yield stress and viscosity were determined using the Casson model Equation (1), fitting the descending flow curves:

(1) $$\sqrt{\tau }=\sqrt{\tau_{0}}+\sqrt{{ƞ}_{\infty }}\;.\;\sqrt{ɣ}$$

where τ is the shear stress (Pa), $\tau_{0}$ is the yield stress (Pa), ${ƞ}_{\infty }$ is the viscosity at an infinite shear rate (Pa.s), ɣ is shear rate (s^−1^).

The reaction kinetics of geopolymer were evaluated by isothermal calorimetry in a TAM Air calorimeter (TA Instruments, New Castle, DE, USA). The temperature was kept at 23 °C and measurements were recorded up to 45 h.

The compressive strength of geopolymer pastes was determined at 7 and 28 days, according to ASTM C1231 [41]. Six specimens (Ø20 mm × h 26 mm) were tested for each composition, and mean values were adopted.

The X-Ray diffraction (XRD) was conducted on a Miniflex II Desktop X-Ray Diffractometer (Rigaku, Tokyo, Japan), operating at the following parameters: 30 kV/15 mA, Cu radiation (λ = 1.5406Å), 2θ from 10 to 70°, and 0.02°/sec scanning rate. Fourier-transform infrared spectroscopy (FTIR) of geopolymer was performed in KBr pellets in a Cary 600 Series FTIR Spectrometer (Agilent, Santa Clara, CA, USA), with an analysis range of 500 to 4000 cm^−1^, resolution of 8 cm^−1^, and 64 accumulations. For microstructural analyses, the samples were immersed in isopropanol [42,43] and ground until a size ≤45 µm.

## 3. Results and Discussions

### 3.1. Zeta potential of SCW and CNT Aqueous Dispersions

**Figure 7:**
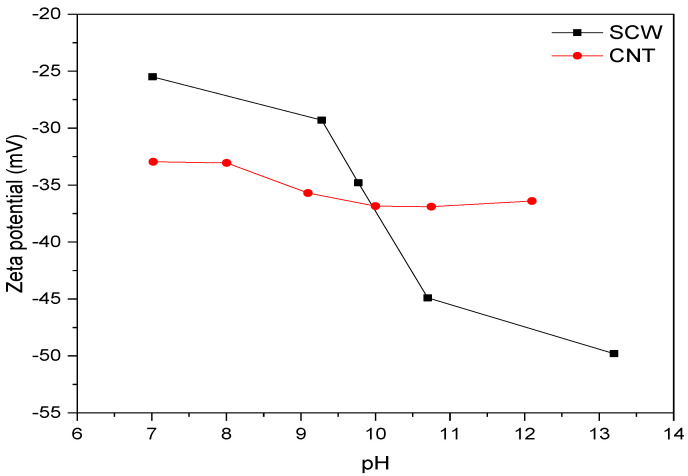
Zeta potential of SCW and CNT aqueous dispersions at different pH values.

The stability of SCW and CNT aqueous dispersions in different pH values was evaluated due to the possibility that their get agglomerates during interaction with the alkaline solution. Thus, the zeta potential (ZP) was determined at different pH values, according to the results presented in Figure 7. The isoelectric point of the CNT and the SCW dispersion occurs in lower pH values than those analyzed. In this context, some authors reported that this point occurs at a pH close to 4 for CNT without treatment [44] and shifts slightly to larger regions (pH ≥ 5) with superficial treatment like NH_2_ or COOH [45]. For SCW, Meguro et al. [15] observed an agglomeration trend of SiC particles (type β) at a pH of 5.0. Other authors indicated an agglomeration at a pH of 5.3 [46] or 3.6 [47]. Thus, considering the studies previously mentioned and that suspensions recorded absolute zeta potential values equal to or greater than 25 mV [48] between pH range of 7–13, the SCW and CNT possibly have electrostatic stability, mainly close to the pH value of A.S. (~14). These results suggest no tendency of CNT and SCW particles to agglomerate.

### 3.2. Rheological Tests

**Figure 8:**
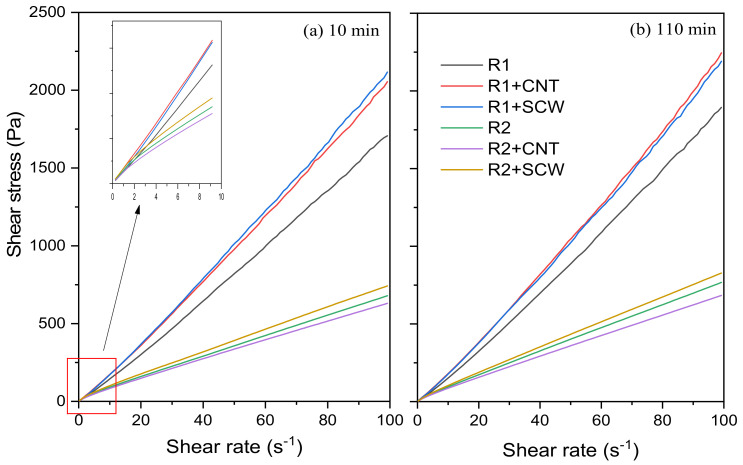
Shear stress of mixtures during: (**a**) 10 min; and (**b**) 110 min after contact between A.S and precursor.

The fresh state of geopolymer pastes was assessed through rotational rheometry. Figure 8 shows the shear stress vs. shear rate curves of the compositions evaluated after 10 and 110 min of the contact of the precursor with the A.S. to exemplify the rheological behavior of metakaolin-based geopolymers. Pastes based on MK1 (R1, R1 + CNT, and R1 + SCW) were more viscous and difficult to spread than those made with MK2 (R2, R2 + CNT, R2 + SCW). R1 group and its derivatives have a shear stress range between 1.70–2.11 Pa, after 10 min. This group registered a slight increase of 11% after 110 min for R1 geopolymer, while the compositions incorporating CNT and SCW increased 9.30% and 3.49%, respectively. In contrast, the R2 group and its derivatives have a lower shear stress range, values between 630 and 741 Pa, 60.21% less than the R1 groups. This trend will be further discussed.

**Figure 9:**
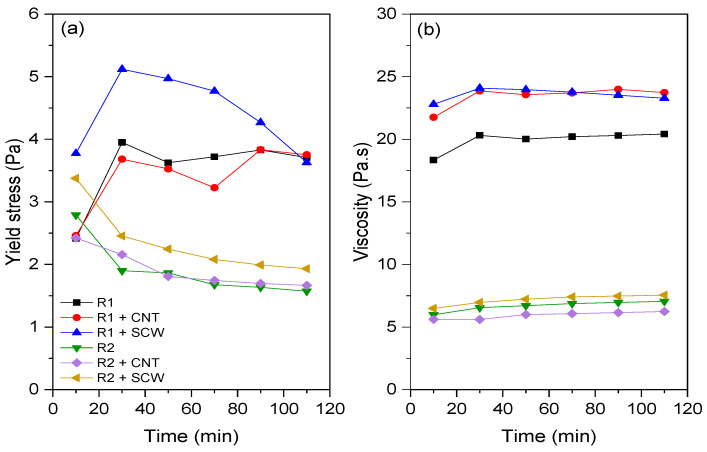
Rheological parameters by Casson model for all groups: (**a**) yield stress; (**b**) viscosity.

Flow curves (i.e., shear stress vs. shear rate) were fitted using Bingham (B), modified Bingham (MB), Herschel–Bulkley (HB), and Casson models (see Supplementary Material). Except for the Casson model, the other evaluated models resulted in some negative values of the rheological parameters (e.g., yield stress and viscosity). Thus, although B, MB, and HB models are the most widely applied models to describe the rheological behavior of geopolymers [49], in this study the flow curves of systems evaluated were fitted to the Casson model, showing a good correlation (R^2^ ≥ 0.9994). Alvi et al. [50] also employed the Casson model to assess the rheological properties of rock-based geopolymers. The yield stress and viscosity values of geopolymer pastes over time are represented in Figure 9. The fresh-state behavior of MK-based geopolymers evaluated in this study agrees with the rotational rheometry results described in the literature. Geopolymer has lower yield stress and higher viscosity values than ordinary Portland cement [51]. Thus, depending on these characteristics in the fresh state, they can be used for self-compacting or self-leveling applications since the low yield stress allows the material to flow, and the high viscosity enhances de stability during casting [52].

Unlike what occurs for materials based on Portland cement, which generally present an approximately linear evolution of yield stress in the first 1 h of hydration [53,54], the geopolymer matrices did not show a general trend. R1 group showed an increase in yield stress between 10 and 30 min; after that, this value tended to stabilize up to 110 min. The equivalent viscosity remained practically constant between 10 and 110 min. For the R2 group, a contrary trend was observed. There was a reduction in the yield stress values over time. While the viscosity also remained practically unchanged. Similar results were reported by Li et al. [55] regarding the rheological behavior of fly-ash geopolymer. The authors also observed constant yield stress and plastic values between 7 and 27 min.

According to Favier et al. [52], in metakaolin-based geopolymer the viscosity is essentially governed by the high viscosity of A.S. and not by the contact between precursor particles. Nevertheless, the same A.S. was used in both systems evaluated (i.e., R1 e R2). Thus, the differences in yield stress and viscosity can be associated with metakaolin characteristics. As expected, the R1 groups (composed of MK1) exhibited higher yield stress and viscosity values than the R2 geopolymers group. This can be related to the higher SSA of MK1 (~235% compared to MK2) and its shape with stacked units (Figure 2). According to Zhou et al. [56], the stacked multilayer structure of MK1 adsorbed a large amount of water, resulting in a higher viscosity coefficient.

The CNTs and SCWs had a more pronounced effect on the rheological properties (e.g., yield stress and viscosity) of the R1-based system compared to the R2-group. Concerning the viscosity, both additions increased the equivalent viscosity by up to 24.3% compared to the reference group (R1). Regarding the yield stress, only the SCW incorporation increased this value for R1 group (by up to 56.0%), while CNT did not affect the shear stress required for the material to flow (i.e., yield stress). However, for the R2-based system, the conditions are different; the presence of CNTs did not significantly affect the yield stress and viscosity, while the SCW incorporation slightly increased the yield stress regardless of the evaluated time. Overall, this behavior is contrary to what was observed regarding incorporating nanomaterials in Portland cement matrices, i.e., the influence of nanomaterials is not so expressive on both rheological parameters of geopolymers. For instance, a previous study identified that the incorporation of CNT caused increases of up to 550.0% and 666.0% in the yield stress and viscosity of CNT-cementitious composites compared to the plain matrix [57]. In geopolymeric systems, Taborda-Barraza et al. [40] reported increases of approximately 40 and 12.2% in the yield stress and viscosity, respectively, with the incorporation of a SCW content of 0.2 wt.%.

Regarding the CNT addition in geopolymers systems, Jindal and Sharma [31], mention that the studies that evaluated the fresh state of CNT-geopolymer composites are still limited. One of the only studies found in the literature is by Alvi et al. [50], where the authors observed that a plain rock-based geopolymer showed yield stress of 0.015 Pa and a viscosity of 0.23 Pa.s, at a temperature of 30 °C. While the hydroxyl-functionalized CNT addition results in values of 0.25 Pa and 0.29 Pa.s, respectively, compared to the plain geopolymer.

### 3.3. Isothermal Calorimetry

**Figure 10:**
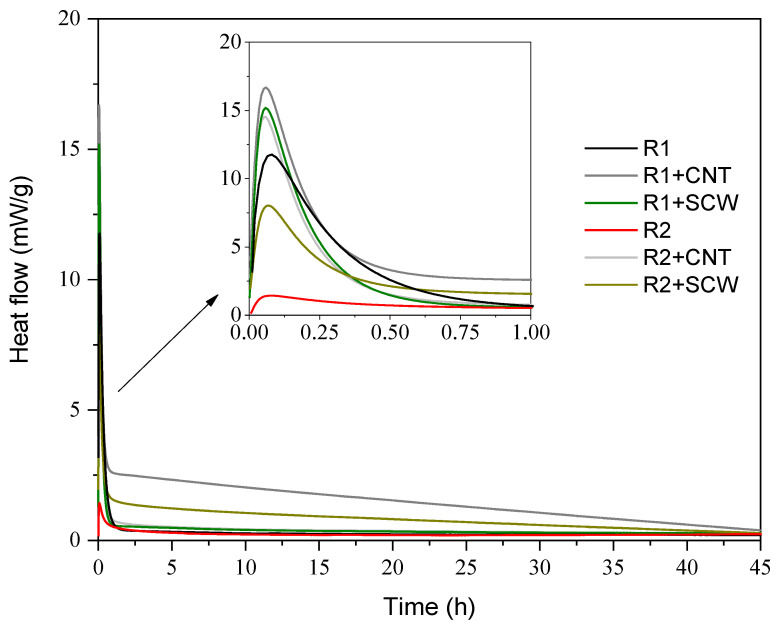
Heat flow and of geopolymers for 45 h.

Figure 10 shows the heat flow and the cumulative heat for all groups. There are few discussions concerning a matrix exclusively based on MK and with the addition of nanomaterials. Nevertheless, the behavior of the heat flow curve is consistent with the literature: systems based on MK with higher Ms (SiO_2_/Na_2_O) of A.S. do not develop a second peak at during the next 42 h [58,59,60], and groups without nanomaterials get thermal stability closed to 10 h after samples preparations. A single exothermic peak is observed for all evaluated systems, mainly associated with the wetting and dissolution of the precursor on A.S. [61,62]. R1-based groups indicate greater intensity in heat flow, which is related to the reactivity of MK1 being greater than MK2. However, the surface energy added to the system by introducing nanoparticles cannot be ignored, which also changes the focal points of the dissolution process.

The presence of CNT and SCW modifies geopolymer reaction kinetics; this fact is evidenced by the modification of the heat flow peaks and time until the heat flow gets thermal stability. The CNT and SCW addition increased the main heat flow peak, suggesting that their presence shifted the energy diffusion rate of the particles interacting with the S.A [63]. Other authors had already registered this behavior with other types of nanomaterials [64,65,66]. Accordingly, the addition of CNT causes the most significant changes, while SCW is between the reference and CNT groups for both systems. This can be justified by the parameter of SSA that is higher for CNT than SCW.

Additionally, CNTs have a functionalized surface that would cause greater chemical affinity between the medium and possibly accelerate the reactions in the first hours. The functional groups on the nanomaterials surface can interact with monovalent ions, such as sodium (−1.77 eV) present in A.S [67]. Long et al. [68] and Taborda-Barraza [40] also registered an increased on reactions peaks with the nanomaterials incorporation, which suggests that nanomaterials provided nucleation’s sites, and depending on their SSA, can modify the final reaction degree.

### 3.4. Compressive Strength

**Figure 11:**
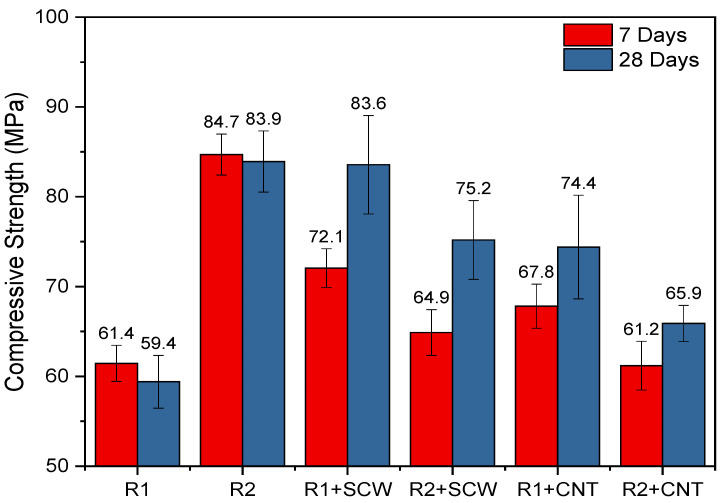
Compressive strength of pastes evaluated on 7 and 28 days.

The mechanical performance of the systems was characterized by the compressive strength at 7 and 28 days, as shown in Figure 11. Visually, it can be identified that even over time, the group’s reference already has mechanical stability that does not generate differences in the values at evaluated ages. It was expected that the MK2-based system had lower mechanical performance than MK1, due to the degree of reactivity and composition associated with the precursor, as previously discussed. Kuenzel et al. [69] evaluated various types of MK under the same activation conditions verifying higher resistance for the one with greater cumulative heat at the end of the evaluation period. However, this did not happen in this study. Thus, the molar ratios of R1 and R2-bases systems were compared to understand this trend.

As seen in Table 4, R2-based systems have slightly higher ratios for SiO_2_/Al_2_O_3_ and Na_2_O/Al_2_O_3_ than R1 group, which can promote more reactions of structuration between chains and progressively results in a higher mechanical performance of R2, even though MK2 also has a lower SSA than MK1 [70,71]. Also, according to the reactivity test (Figure 4), MK2 exhibited a higher reactivity during the first 80 h and after that MK1 increased its performance. This also suggests that R1 (composed by MK1) can still develop more strength over time. Nath et al. [72], also observed significant increases in the compressive strength of geopolymer concretes between 28 and 90 days, reducing the percentage difference between the systems [73,74]; this can be possible on R1-groups considering the reactive nature of MK1.

The R1 and R2 systems containing SCW and CNT registered different performances. As shown in Figure 11, the SCW and CNT incorporation cause positive effects (increase) exclusively on R1- based groups, while for R2-based groups, all addition causes reductions in compressive strength. For the 28 days, the highest increment reached 40.7%, with SCW addition. In the R2-based group, the additions lead to a drop in strength, which allows us to infer that this matrix may have reached the best performance without nanomaterial incorporation. Its presence ended up harming the continuity of the matrix, which promotes significant reductions, up to 27.76% when CNTs are added or up to 23.39% when SCWs are added, from the first 7 days.

Similar conditions have been observed when different types of nanomaterials are intended to reinforce ceramic and polymeric matrices; the additions do not always improve the matrix’s mechanical performance [75,76]. The performance would be conditioned to the type of nanomaterial and the individual performance of the matrix, which, being of high strength, may be harmed by the nanomaterial incorporation. Some theoretical studies stand out: understanding the interphases nano/composite is essential [77].

### 3.5. Microestruture: XRD Results and FTIR

**Figure 12:**
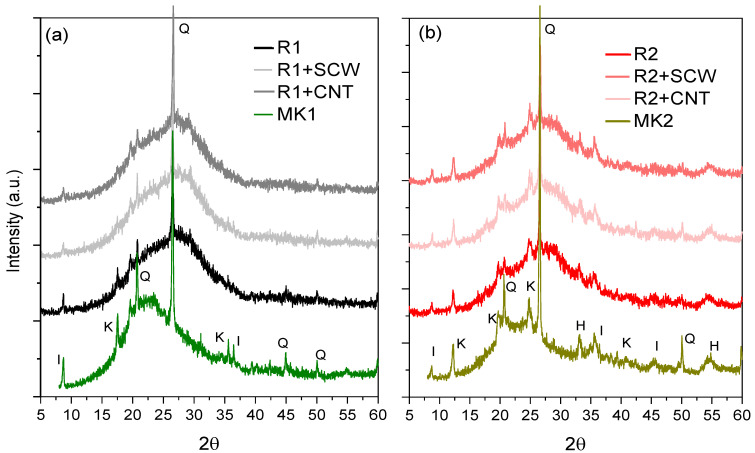
XRD spectrum of groups based on: (**a**) MK1; (**b**) MK2. Specifications: I—Ilite, K—kaolinite, Q—quartz, and H—hematite.

Figure 12 shows that the amorphous halo between 15° and 35° 2θ of the precursor material was shifted at 28 days to larger regions, which can be translated as chemical transformations in the system. At the same time, the higher height visualization of the same halo for MK1 compared to MK2 also suggests, according to several authors, the formation of more amorphous reaction products [78,79] which is consistent with the reactivity of MK1 compared to MK2 (Figure 4). Based on the above, the MK2-based system is not more resistant due to the formation of more reaction products but because of the possible formation of longer polymeric chains, supported by the variations recorded in the molar ratios of the MK2-based system, where Na_2_O/Al_2_O_3_ and SiO_2_/Al_2_O_3_ registered values greater than MK1- based system.

**Figure 13:**
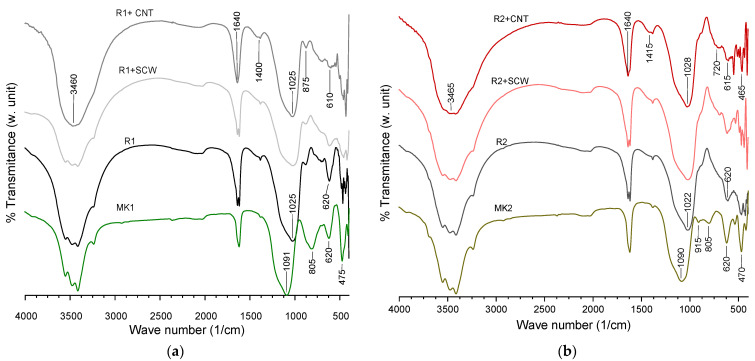
FTIR Spectrum of mixtures at 28 days: (**a**) MK1-based groups, (**b**) MK2-based groups.

Complementary, the FTIR spectrum, observed in Figure 13, registers the shift on the precursor after activation. The principal bands were identified and compared on Table 5. Peaks associated with the incorporation of any nanomaterial could not be identified. The precursors have vibrations bands at 470, 805 and 915 cm^−1^ related to Si-O, Al-O e Si-O-Si/Al chemical bonds, respectively. That shifted their intensity or disappeared. In contrast, the band around 1091 cm^−1^ loses intensity that moves to lower regions. All these transformations obey the breakages and restructure micro-structural on MK after activation. The main similarity in the groups is the displacement to smaller regions in the activated material compared with the original precursor.

Nevertheless, R1-groups registered a small band related to possible Si-OH, which could infer that they still have unreacted material to accompany mechanical resistance growth after the evaluated age. In the same way, the presence of C-O band looks more intense in groups containing CNT, but this is not significant to form a phase on the XRD spectrum so that it can be discarded as carbonates attack.

## 4. Conclusions

This study evaluated the incorporation of CNT and SCW in the fresh and hardening states of two types of metakaolin-based geopolymers paste, through rotational rheometry, isothermal calorimetry conduction and compressive strength.

Both systems showed low yield stress due to a high Na_2_SiO_3_/NaOH ratio and, concomitantly, high equivalent viscosity values. The incorporation of CNT and SCW did not cause any difference in the yield stress and viscosity for R2-based geopolymers. In contrast, the viscosity and yield stress increased 20.0 and 56.0% for R1-based systems.

The mechanical reinforcement of MK-based geopolymers with CNT or SCW can be achieved; nonetheless, this is possibly conditionate to the strength characteristics of the system. For instance, maximum compressive strength of 83.56 MPa was achieved for the R1+SCW combination (an enhancement of 40.7% compared to the reference geopolymer). The nanomaterials incorporation improves an average mechanical performance matrix. However, matrices with high mechanical performance will compromise this parameter because the addition possibly generates discontinuities in the matrix and can act as points to crack propagation or isolated regions.

Even when in this study it was verified that CNT and SCW nanomaterials can improve the compressive strength in one group evaluated, other kinds of tests can be applied to evaluate the mechanical performance such as flexural strength and define dynamics modulus and porous distribution as complementary information.

## Figures and Tables

**Table 1:** Chemical composition MK1 and MK2.

Compound	SiO_2_	Al_2_O_3_	Fe_2_O_3_	K_2_O	MnO	TiO_2_	Fire Lost	SSA (m^2^/g)
MK1	56.27	42.40	0.44	0.67	0.01	0.02	0.59	29.02
MK2	51.28	38.27	7.18	1.04	0.17	1.63	3.00	8.65

**Table 2:** CNT characteristics.

Inside Diameter (nm)	Outside Diameter (nm)	Length (µm)	SSA (m^2^/g)	Purity	-COOH Content (%)
5–10	20–30	10–30	182.1	95%	1.9–2.1%

**Table 3:** SCW characteristics.

Diameter (µm)	Length (µm)	SSA (m^2^/g)	Carbon Free	Type Cristal
0.1–2.5	2–50	12.55	≤0.05%	β

**Table 4:** Molar ratios from each mixture and additions content incorporated.

Name	Addition (gr)	SiO_2_/Al_2_O_3_	Na_2_O/Al_2_O_3_	Na_2_O/SiO_2_	H_2_O/Na_2_O
R1 (MK1 + AS)	0.00	3.70	0.68	0.18	14.75
R2 (MK2 + AS)	0.00	3.88	0.76	0.20	
R1 + NW	0.20	3.70	0.68	0.18	
R2 + NW	0.20	3.88	0.76	0.20	
R1 + CNT	0.20	3.70	0.68	0.18	
R2 + CNT	0.20	3.88	0.76	0.20	

**Table 5:** Specifications of vibration band for R1 and R2 based groups.

Type of Vibration	Wavenumber (cm^−1^)	References	MK1	R1	R1 + SCW	R1 + CNT	MK2	R2	R2 + SCW	R2 + CNT
O-H stretching, H-O-H bending	3460–3465,1640	[31,38,55]	+	+	+	+	+	+	+	+
C-O asymmetric stretching	1400–1415, 875	[64,80]	-	+	+	++	+	+	+	++
Si-O-T asymmetric stretching (T = Al, Si)	1200–900	[29,36,51,52,54,67,77]	+	+	+	+	+	+	+	+
Al-O stretching	800–805, 620	[71]	++	++	+	+	+/++	-/+	-/+	-/+
Si-OH bending	865–875	[58]	-	+	+	++	-	-	-	-
Si-O-Si, Al-O-Si symmetric stretching	465–475	[55,71]	++	+	+	+	++	+	+	+

Legend: +: Present low intensity, ++: Present high intensity, -: Absent.

## Data Availability

Not applicable.

## Supplementary Materials

The following supporting information can be downloaded at: https://www.mdpi.com/article/10.3390/ma15176099/s1, this document has information about models parameters used to characterize the rheological behavior.
